# “*Candidatus* Fokinia solitaria”, a Novel “Stand-Alone” Symbiotic Lineage of *Midichloriaceae* (*Rickettsiales*)

**DOI:** 10.1371/journal.pone.0145743

**Published:** 2016-01-05

**Authors:** Franziska Szokoli, Elena Sabaneyeva, Michele Castelli, Sascha Krenek, Martina Schrallhammer, Carlos A. G. Soares, Inacio D. da Silva-Neto, Thomas U. Berendonk, Giulio Petroni

**Affiliations:** 1 Institut für Hydrobiologie, Technische Universität Dresden, Dresden, Germany; 2 Dipartimento di Biologia, Università di Pisa, Pisa, Italy; 3 Department of Cytology and Histology, St. Petersburg State University, St. Petersburg, Russia; 4 Mikrobiologie, Biologisches Institut II, Albert-Ludwigs Universität Freiburg, Freiburg, Germany; 5 Departamento de Genética, Universidade Federal do Rio de Janeiro, Rio de Janeiro, Brazil; 6 Departamento de Zoologia, Universidade Federal do Rio de Janeiro, Rio de Janeiro, Brazil; University of Poitiers, FRANCE

## Abstract

Recently, the family *Midichloriaceae* has been described within the bacterial order *Rickettsiales*. It includes a variety of bacterial endosymbionts detected in different metazoan host species belonging to Placozoa, Cnidaria, Arthropoda and Vertebrata. Representatives of *Midichloriaceae* are also considered possible etiological agents of certain animal diseases. *Midichloriaceae* have been found also in protists like ciliates and amoebae. The present work describes a new bacterial endosymbiont, “*Candidatus* Fokinia solitaria”, retrieved from three different strains of a novel *Paramecium* species isolated from a wastewater treatment plant in Rio de Janeiro (Brazil). Symbionts were characterized through the full-cycle rRNA approach: SSU rRNA gene sequencing and fluorescence *in situ* hybridization (FISH) with three species-specific oligonucleotide probes. In electron micrographs, the tiny rod-shaped endosymbionts (1.2 x 0.25–0.35 μm in size) were not surrounded by a symbiontophorous vacuole and were located in the peripheral host cytoplasm, stratified in the host cortex in between the trichocysts or just below them. Frequently, they occurred inside autolysosomes. Phylogenetic analyses of *Midichloriaceae* apparently show different evolutionary pathways within the family. Some genera, such as “*Ca*. Midichloria” and “*Ca*. Lariskella”, have been retrieved frequently and independently in different hosts and environmental surveys. On the contrary, others, such as *Lyticum*, “*Ca*. Anadelfobacter”, “*Ca*. Defluviella” and the presently described “*Ca*. Fokinia solitaria”, have been found only occasionally and associated to specific host species. These last are the only representatives in their own branches thus far. Present data do not allow to infer whether these genera, which we named “stand-alone lineages”, are an indication of poorly sampled organisms, thus underrepresented in GenBank, or represent fast evolving, highly adapted evolutionary lineages.

## Introduction

The order *Rickettsiales* belongs to the *Alphaproteobacteria* and exclusively comprises obligate intracellular bacteria including causative agents for serious human diseases, such as *Rickettsia rickettsii* (Rocky Mountain spotted fever), and *Rickettsia prowazekii* (epidemic typhus) [1–3]. For many years, it was mainly the pathogenicity of species such as *Rickettsia*, *Anaplasma* and *Ehrlichia* that stirred up the interest in this group. Later, the discovery of their close relationship to mitochondria fueled speculations on their phylogeny and evolution [4–6]. Studies on so-called “neglected *Rickettsiaceae*” or *Rickettsia*-like organisms (RLO) inhabiting non-haematophagous hosts opened further perspectives in this field, both from the evolutionary and ecological points of view [7–10]. Studying the biodiversity of *Rickettsiales* will not only provide missing links needed to resolve the intricate evolutionary patterns within *Rickettsiales per se* and enlighten their role as partners in numerous symbiotic systems, but also broaden our knowledge of host-symbiont interaction and its development during evolution.

At present the order *Rickettsiales* comprises the families [11]: i) *Rickettsiaceae*, with the genera *Rickettsia*, *Orientia*, *Occidentia* [12], “*Candidatus* (*Ca*.) Megaira” [10], “*Ca*. Cryptoprodotis” [8], “*Ca*. Arcanobacter” [13], “*Ca*. Trichorickettsia” and “*Ca*. Gigarickettsia” [14]; ii) *Anaplasmataceae*, with the genera *Anaplasma*, *Wolbachia*, *Ehrlichia*, *Neorickettsia* [15,16], *Aegyptianella* [17], “*Ca*. Neoehrlichia” [18], “*Ca*. Xenohaliotis” [19], and “*Ca*. Xenolissoclinum” [20]; and iii) the newly described “*Ca*. Midichloriaceae” (*Midichloriaceae* from now on). Recently, *Midichloriaceae* have been recognized as a clade or even a putative family by several authors [3,21–25] and finally received their formal family description by Montagna and colleagues [26]. The status of a fourth family, *Holosporaceae*, is presently debated. It fell at the base of *Rickettsiales* evolution in several phylogenetic trees based on SSU rRNA gene analyses (e.g. [25,27–30]), and even on concatenated protein coding genes [25]. However, other recent studies that consider LSU rRNA and/or different sets of protein coding genes seem to contradict this view, suggesting alternative placements of *Holosporaceae* within *Alphaproteobacteria* [11,31–33]. Whatever the position of *Holosporaceae* is, it does not affect the monophyletic evolutionary status of the three families *Rickettsiaceae*, *Anaplasmataceae* and *Midichloriaceae*, defined in all studies addressing their phylogeny (e.g. [11,26,30]). Therefore, *Holosporaceae* will not be discussed here.

The families of the order *Rickettsiales* show differences in their host range. Up to now, members of the *Anaplasmataceae* have been only detected in animals (Metazoa), thus suggesting a certain host specificity (reviewed in [16]). The family *Rickettsiaceae* was considered to inhabit only arthropods and vertebrates as alternating hosts. Rather unexpectedly, members of this family, including species showing no pathogenicity to vertebrates [7], have been recently detected in most eukaryotic super-groups as defined by Adl and colleagues [34]. *Rickettsia*-like endosymbionts occur in Opisthokonta, such as Metazoa (e.g. in leeches [35]) and Holomycota (e.g. *Nuclearia* [36]); in Archaeplastida, such as green algae [37–39] and higher plants [40]; in SAR (Stramenopiles, Alveolata, Rhizaria) host organisms, such as Alveolata, mainly ciliates [8,10,14] and Rhizaria [41]; in Excavata, such as euglenozoans [42,43]. In particular, “*Ca*. Megaira polyxenophila” shows an exceptionally broad host range inhabiting different ciliates [10,44,45], cnidarians [46,47] and green algae [37,39], indicating the possibility of horizontal transfer.

Similarly, the recently described family *Midichloriaceae*, with “*Ca*. Midichloria” as a type genus, revealed a striking biodiversity in the last years. *Midichloriaceae* as a whole show a wide host range. They can invade not only different arthropods including ticks, fleas, bed bugs, seed bugs and gadflies [21,48–51], but also other metazoan species such as *Trichoplax adhaerens* [25] and cnidarians [46,52]. Associations to fish [53,54] and mammals [55,56], including humans [24,51,57], were detected as well. Moreover, they have been found in Amoebozoa [58] and Ciliophora [22,59,60]. Indeed, they have been detected in organisms belonging to different eukaryotic super-groups (for review see [25,26]) but up to now, there are no records from Archaeplastida and Excavata.

Representative hosts of both, *Rickettsiaceae* and *Midichloriaceae*, are found at various trophic levels of the food chain, suggesting the theoretical possibility of horizontal transfer of the bacteria from one host to another by trophic interaction. Though not yet proven for all *Rickettsiales*, recent findings support the idea of possible host shifts in some *Rickettsiaceae* [9,10]. Data on recently described members of *Midichloriaceae* (i.e. “*Ca*. Defluviella procrastinata” in *Paramecium nephridiatum* and “*Ca*. Cyrtobacter zanobii” in *Euplotes aediculatus*) support the notion of the independent establishment of different symbiotic systems involving *Midichloriaceae* and ciliates during evolution [32,60,61]. Protists may have served as a source of infection for other organisms in aquatic environments, and may have facilitated the later transfer of *Rickettsiaceae* and *Midichloriaceae* to terrestrial habitats by arthropods. Taking into account the frequent occurrence of *Midichloriaceae* in haematophagous ticks [21,23,62–66] and bed bugs [50], it is only a little step up to the tick’s or bug’s victim, a potential vertebrate host. This putative course of host range expansion is presently supported by a growing evidence for potential infectivity of *Midichloriaceae* towards vertebrates [53,56,57]. Thus, studying this group may result in finding new potential pathogens of humans and economically important vertebrate species.

In the present study we provide an ultrastructural, molecular and phylogenetic description of a novel bacterial endosymbiont representing a new solitary branch within the *Midichloriaceae* family. It was recently discovered in a *Paramecium* species collected from a wastewater treatment plant in Rio de Janeiro (Brazil). According to the taxonomic rules for uncultivable bacteria [67], we propose to name the endosymbiont species “*Ca*. Fokinia solitaria”. New insights into the evolutionary pattern of *Rickettsia*-Like Organisms are also discussed.

## Materials and Methods

### Host isolation, cultivation and identification

The *Paramecium* strains Rio ETE_ALG 3VII, 3IX, 3X and 3XI were isolated from the wastewater treatment plant Estação de Tratamento de Esgoto Alegria (22°52'16"S 43°13'44"W, Rio de Janeiro, Brazil) in February 2012. Sampling permission was provided by the State water and sewage company CEDAE (Companhia Estadual de Águas e Esgotos do Rio de Janeiro). Monoclonal cultures (strains) were established and maintained at 22 ± 1°C in 0.25% Cerophyl-medium inoculated with *Enterobacter aerogenes* [68]. According to morphological features [69] and eukaryotic SSU rRNA gene sequencing [70] the host was identified at genus level. As strain Rio ETE_ALG 3XI lost its endosymbionts after few generations of cultivation, bacterial SSU rRNA gene sequence and TEM observation were not obtained for this strain.

### DNA extraction

Total DNA extraction was performed according to the following protocol: approximately 50 cells were washed by six successive transfers into sterile mineral water (Volvic®, Danone Waters, Paris, France); *Paramecium* cells were starved overnight in sterile Volvic water, washed again six times to minimize bacterial contamination and fixed in 70% ethanol. DNA was extracted applying the NucleoSpin® Plant DNA Extraction Kit (Macherey-Nagel GmbH & Co. KG, Düren NRW, Germany), following the CTAB protocol for mycelium.

### Molecular characterization

For the molecular characterization of the endosymbiont, bacterial SSU rRNA genes were amplified with the *Alphaproteobacteria* specific forward primer 16Sα_F19b 5'-CCTGGCTCAGAACGAACG-3' [71] and the universal bacterial reverse primer 16S_R1522a 5'- GGAGGTGATCCAGCCGCA -3' [71] using a touchdown PCR with annealing temperatures of 58°C (30 sec, 5 cycles), 54°C (30 sec, 10 cycles) and 50°C (30 sec, 25 cycles). The reaction was carried out in a C1000 Thermal cycler form Bio-Rad Laboratories (Hercules, CA, USA). The obtained PCR products were purified with the EuroGold CyclePure Kit (EuroClone S.p.A. Headquarters & Marketing, Pero Milano, Italy) and sequenced using the internal primers 16S F343 ND 5’-TACGGGAGGCAGCAG-3’, 16S R515 ND 5’-ACCGCGGCTGCTGGCAC-3’ and 16S F785 ND 5’-GGATTAGATACCCTGGTA-3’ [71] at GATC Biotech AG (Konstanz, Germany).

### Species-specific probe design

In order to verify that the sequenced bacterial SSU rRNA gene amplicon derived from the endosymbiont, three species-specific probes were designed: Fokinia_198 5'-CTTGTAGTGACATTGCTGC-3' (Alexa488-labeled, T_m_ = 54.5°C), Fokinia_434 5'-ATTATCATCCCTACCAAAAGAG-3' (Cy3-labeled, T_m_ = 54.7°C) and Fokinia_1250 5'-ACCCTGTTGCAGCCTTCT-3' (Cy3-labeled, T_m_ = 56.0°C). T_m_ was determined by Eurofins GMBH (Ebersberg, Germany) that synthetized the probes. FISH experiments using one of the species-specific probes in combination with the almost universal eubacterial probe EUB338 (either FITC- or Cy3-labeled [72]) were performed. The newly designed probes were tested at different formamide concentrations ranging from 0% up to 50%; their specificity was *in silico* determined using the TestProbe tool 3.0 (SILVA rRNA database project [73]) and the probe match tool of the Ribosomal Database Project (RDP [74]) allowing 0, 1 or 2 mismatches (Table 1). Finally, they have been uploaded to ProbeBase [75] and figshare (DOI: 10.6084/m9.figshare.2008524).

**Table 1:** *In silico* matching of the species-specific probes Fokinia_198, Fokinia_434 and Fokinia_1250 against bacterial SSU rRNA gene sequences available from RDP (release 11, update 4) and SILVA (release 123) databases. Number of sequences in the corresponding database was 3,333,501 (RDP) or 1,756,783 (SILVA). “mism” stands for “mismatch(es)”. Reported are the number of sequences (“hits”) which theoretically hybridize with the probe allowing for the given number of mismatches.

Species-specific probe	RDP			SILVA		
	0 mism	1 mism	2 mism	0 mism	1 mism	2 mism
Fokinia_198	0 hits	0 hits	7 hits	0 hits	0 hits	0 hits
Fokinia_434	0 hits	84 hits	1,540 hits	0 hits	18 hits	437 hits
Fokinia_1250	0 hits	0 hits	13 hits	0 hits	0 hits	4 hits

### Fluorescence *in situ* hybridization (FISH)

FISH experiments were performed to detect the presence of endosymbiotic bacteria. At least 20 cells were washed three times in sterile Volvic and placed on SuperFrost Ultra Plus® slides (Gerhard Menzel GmbH, Braunschweig, Germany). Cells were fixed with 2% paraformaldehyde (PFA), dehydrated in an ethanol gradient and air-dried. Fixed cells were covered with hybridization buffer [76] containing recommended formamide concentration and 10 ng/μL of each probe. Slides were incubated overnight at 46°C in order to increase the accessibility of the bacterial SSU rRNA [77]. The next day, after washing for 20 min at 48°C, slides were air dried, mounted with CitiFluor^TM^ AF1 (Citifluor Ltd, London, Great Britain) containing DAPI, and examined using the fluorescence microscope Nikon Eclipse Ti (Nikon Corporation, Tokyo, Japan).

Alternatively, to reduce autofluorescence background signal, cells were incubated for 30 minutes at 4°C in 2% PFA in depression slides, transferred to microscope slides and incubated again for 30 minutes at 4°C. The surplus liquid was removed. One drop of ice-cold 70% methanol was added, immediately removed and the slides were transferred into a washing chamber filled with 2x PBS at room temperature. Hybridization was performed applying 10 ng/μL of each probe in the hybridization buffer containing optimal formamide concentration (see results). The slides were incubated at 46°C in a humid chamber for 1.5–2 hours, followed by two washing steps in washing buffer [76] for 30 minutes at 48°C. During the whole procedure cells were prevented from drying. Finally, the cells were covered with Mowiol (Calbiochem®, Merck KGaA, Darmstadt, Germany) containing PPD and DAPI according to manufacturer protocol. Images were obtained with a Leica TCS SPE confocal laser scanning microscope (Leica Microsystems GmbH, Wetzlar, Germany).

The used probes were EUB338 (5’-GCTGCCTCCCGTAGGAGT-3’, Cy3-labeled [72]), the *Alphaproteobacteria*-specific probe ALF1b (5’-CGTTCGYTCTGAGCCAG-3’, 6-FAM-labeled [76]) and the specifically designed ones.

### Phylogenetic analysis

The obtained bacterial SSU rRNA gene sequence of “*Ca*. Fokinia solitaria” was aligned with the automatic aligner of the ARB software package version 5.2 [78] together with 22 closely related sequences of the *Midichloriaceae* family, 17 members of the *Anaplasmataceae* and *Rickettsiaceae* and 10 sequences representing the outgroup. The alignment was optimized manually especially focusing on the predicted base pairing of the stem regions, referring to the SSU rRNA structure of *E*. *coli* provided by ARB. The aligned sequences were then trimmed at both ends to the length of the shortest one; gaps were treated as missing data. The resulting alignment (S1 Alignment) contained 1,568 nucleotide columns that were used for phylogenetic inference. The optimal substitution model was selected with jModelTest 2.1 [79] according to the Akaike Information Criterion (AIC). A maximum likelihood (ML) tree was calculated with 1,000 bootstrap pseudoreplicates using the PHYML software version 2.4.5 [80] from the ARB package. Bayesian inference (BI) was performed with MrBayes 3.2 [81], using three runs each with one cold and three heated Monte Carlo Markov chains, with a burn-in of 25%, iterating for 1,000,000 generations (obtained model parameters are shown in S1 Table). The runs were stopped after verifying the average standard deviation of the split frequencies had reached a value 0.01 or below. A similarity matrix [82] was built using the same 1,568 columns employed in phylogenetic reconstructions.

### Transmission Electron Microscopy (TEM)

Ciliates were processed for electron microscopy as described elsewhere [59]. Briefly, the cells were fixed in a mixture of 1.6% PFA and 2.5% glutaraldehyde in 0.1 M phosphate buffer (pH 7.2–7.4) for 1.5 h at room temperature, washed in the same buffer containing sucrose (12.5%) and postfixed in 1.6% OsO_4_ (1 h at 4°C). Then the cells were dehydrated in an ethanol gradient followed by ethanol/acetone (1:1), 100% acetone, and embedded in Epoxy embedding medium (Fluka Chemie AG, St. Gallen, Switzerland). The resin was polymerized according to the manufacturer’s protocol. The blocks were sectioned with a Leica EM UC6 Ultracut. Sections were stained with aqueous 1% uranyl acetate followed by 1% lead citrate.

Negative staining was performed by first washing and starving the cells overnight in distilled water to decrease the abundance of food and environmental bacteria. Single cells were then squashed with the micropipette and the remaining were transferred onto grids covered with the supporting film. Staining was performed using aqueous 1% uranyl acetate. All samples were examined with a JEOL JEM-1400 (JEOL, Ltd., Tokyo, Japan) electron microscope at 90 kV. The images were obtained with an inbuilt digital camera.

### Nucleotide sequence accession number

The sequence obtained from bacterial SSU rRNA gene of the endosymbiont of *Paramecium* clone Rio ETE_ALG 3VII was submitted to the GenBank database (NCBI) under the accession number KM497527 (1,473 bp).

## Results

### Characterization of the host

The *Paramecium* strains isolated from the wastewater samples were submitted to morphological analyses. General morphological features like size, body shape and location of the cytoproct were typical for the *Paramecium caudatum-aurelia* clade. However, species identification proved to be equivocal, suggesting the possibility that we were dealing with a new species. A detailed description of the host including morphometric, ultrastructural, and molecular characterization will be provided in a separate publication.

### Molecular characterization of endosymbiont

Nearly full-length bacterial SSU rRNA gene sequences were obtained for the three strains Rio ETE_ALG 3VII, 3IX and 3X. The sequences were identical, hence, strain Rio ETE_ALG 3VII was used representatively for all three strains (Rio ETE_ALG 3VII: 1,473 bp, GenBank accession: KM497527). NCBI Blastn results against nucleotide collection (nr/nt) showed the highest identity (88.5% and 87.0%, respectively) with an uncultured bacterium from a lake in New York (accession number FJ437943) and “*Ca*. Defluviella procrastinata”, symbiont of *Paramecium nephridiatum*. It is noteworthy that, compared to the other *Midichloriaceae* included in the analysis, the SSU rRNA gene sequences of both “*Ca*. Fokinia solitaria” and “*Ca*. Defluviella procrastinata” had four small insertions (2–13 nucleotides long) in the same positions (76, 94, 200, 216, according to the *E*. *coli* SSU rRNA gene reference numbering). These insertions were paired two by two in the predicted rRNA structure, increasing the length of two stems in regions V1 and V2 respectively. Other two small insertions (4 and 5 nucleotides long, at positions 452 and 476, respectively) were present in “*Ca*. Fokinia solitaria” only, which were predicted to increase the length of a third stem in region V3 of the rRNA molecule.

### FISH experiments

In preliminary FISH experiments, positive signals with both probes (EUB338 and Alf1b) were observed in the cytoplasm of all *Paramecium* strains. Overlapping signals of both probes indicated the presence of endosymbiotic bacteria belonging to *Alphaproteobacteria* in the cell cortex. Bacteria localized in digestive vacuoles (food bacteria) showed positive signals only with probe EUB338.

**Fig 1:**
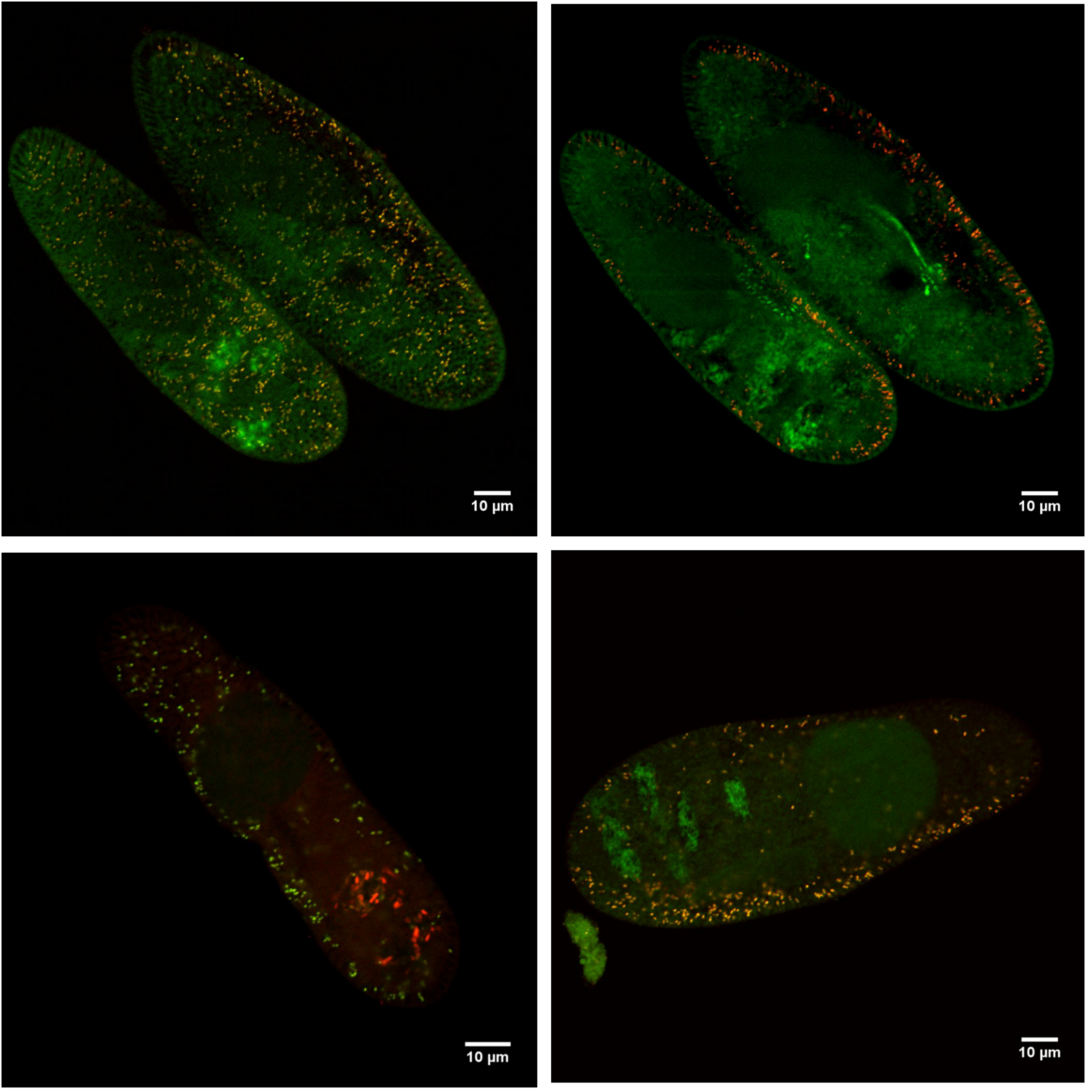
Species-specific *in situ* detection of “*Candidatus* Fokinia solitaria” in *Paramecium* sp. strain Rio ETE ALG 3VII at 15% formamide concentration. Merge of the signals from probes EUB338 (fluorescein-labelled, green signal) and A) species-specific probe Fokinia_434 (Cy3-labelled, red signal), B) alphaproteobacterial probe ALF1b (Cy3), C) species-specific probe Fokinia_198 (labelled with Alexa488, green signal), or D) species-specific probe Fokinia_1250 (Cy3). Stratification of the endosymbiont in section through the host cortex (A, C) and through the inner part of the host cell (B, D). “*Ca*. Fokinia solitaria” appears yellowish. Scale bars: 10 μm.

The designed species-specific probes Fokinia_198, Fokinia_434, and Fokinia_1250 were specifically designed to have a similar and low T_m_ that should have guaranteed good specificity without formamide or with low formamide concentrations. Hybridization experiments with their target organism in a formamide range from 0 to 30%, confirmed the signal intensity was best between 0–15% formamide (Fig 1). Specificity of probes was tested *in silico* against available bacterial SSU rRNA gene sequences (Table 1). The probes Fokinia_198 and Fokinia_1250 showed high specificity even when mismatches were allowed. Probe Fokinia_434, on the other hand, recognized 84 non-target sequences when one mismatch was allowed (Table 1). Experiments with one species-specific probe and either EUB338 or Alf1b clearly showed that “*Ca*. Fokinia solitaria” is the only symbiont residing in the cytoplasm (outside the food-vacuoles) of these host strains (Fig 1).

### Phylogenetic analysis

**Fig 2:**
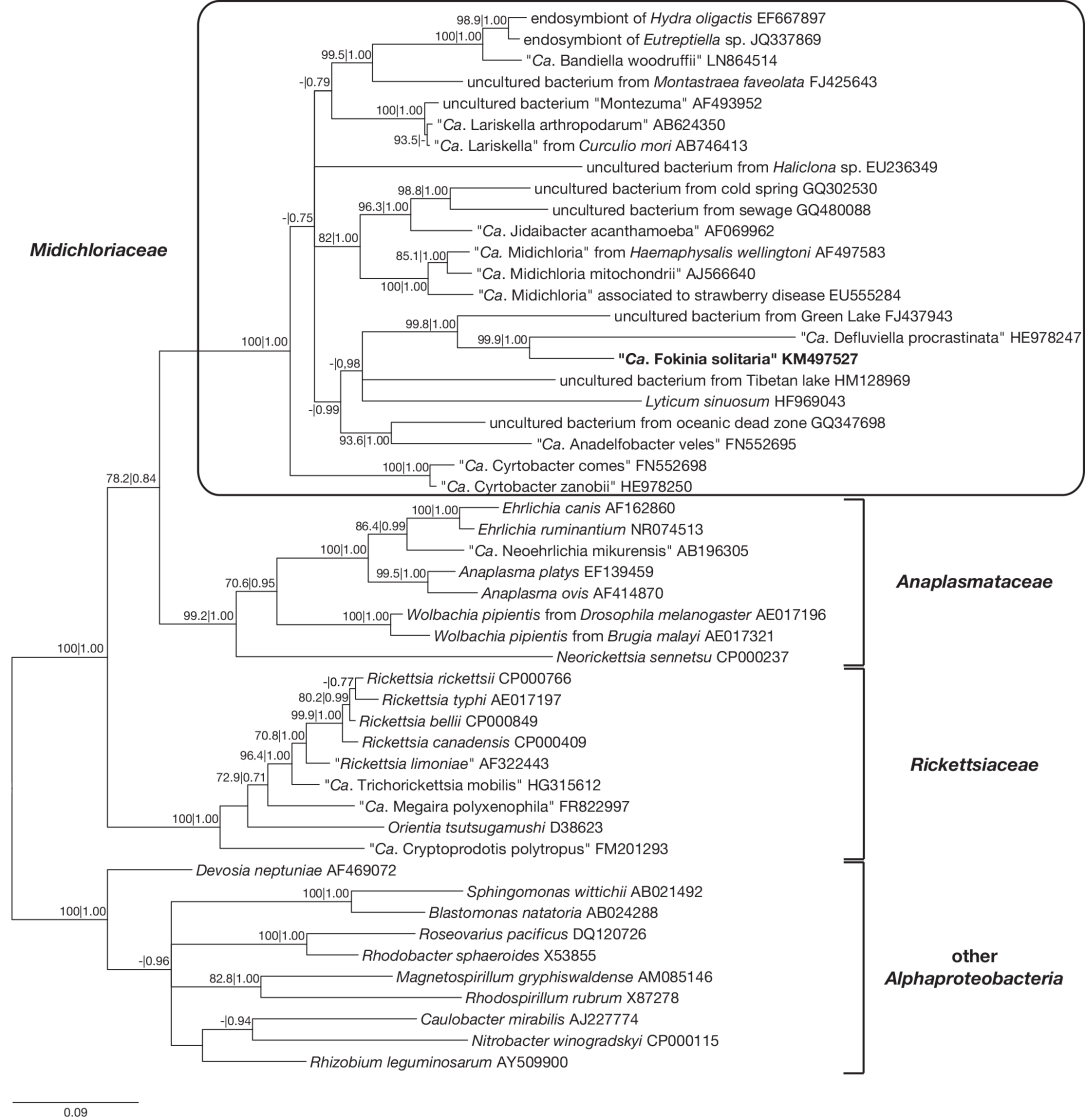
Bayesian inference phylogenetic tree built with MrBayes employing the GTR + I + G model. Numbers indicate bootstrap values inferred after 1,000 pseudoreplicates for maximum likelihood and Bayesian posterior probabilities (values below 70.0% and 0.7 are not shown). The sequence characterized in the present work is reported in bold. Scale bar: 9 nucleotide substitutions per 100 positions. “*Ca*.” stands for “*Candidatus*”.

After the choice of the GTR+I+G substitution model with jModelTest, the ML and BI trees were estimated (Fig 2). The monophyly of the three families *Rickettsiaceae*, *Anaplasmataceae* and *Midichloriaceae* was confirmed with both inference methods, which joined *Midichloriaceae* and *Anaplasmataceae* as the sister group to *Rickettsiaceae*, with bootstrap value 78.2% for ML and posterior probability value 0.80 for BI. Additionally, in both trees several sequences within the *Midichloriaceae* formed well-supported monophyletic clades, like the genera “*Ca*. Midichloria” and “*Ca*. Lariskella”. However, most of the ancient relationships within this family showed comparatively little support and appeared still unresolved, which is in good agreement with literature (e.g. [59,60]). Only the position of “*Ca*. Cyrtobacter” as sister group to all other *Midichloriaceae* was obtained with high support with both inference methods (100% ML; 1.00 BI).

The sequence of “*Ca*. Fokinia solitaria” from *Paramecium* strain Rio ETE_ALG 3VII affiliated to *Midichloriaceae* and was strongly associated (99.9% ML; 1.00 BI) to “*Ca*. Defluviella procrastinata” endosymbiont of *P*. *nephridiatum* (Fig 2), while the identity among them was only 87.0%. The two sequences together were grouped with high support (99.8% ML; 1.00 BI) to the previously mentioned uncultured bacterium from a freshwater lake in New York (FJ437943), which had 88.5% and 84.9% identity with “*Ca*. Fokinia solitaria” and “*Ca*. Defluviella procrastinata”, respectively. The branches leading to the three sequences were long compared to the other *Midichloriaceae* in the obtained phylogenetic tree. Further groupings of the three sequences with the genus *Lyticum*, “*Ca*. Anadelfobacter veles” and other uncultured bacteria were not supported statistically.

### Transmission Electron Microscopy (TEM)

**Fig 3:**
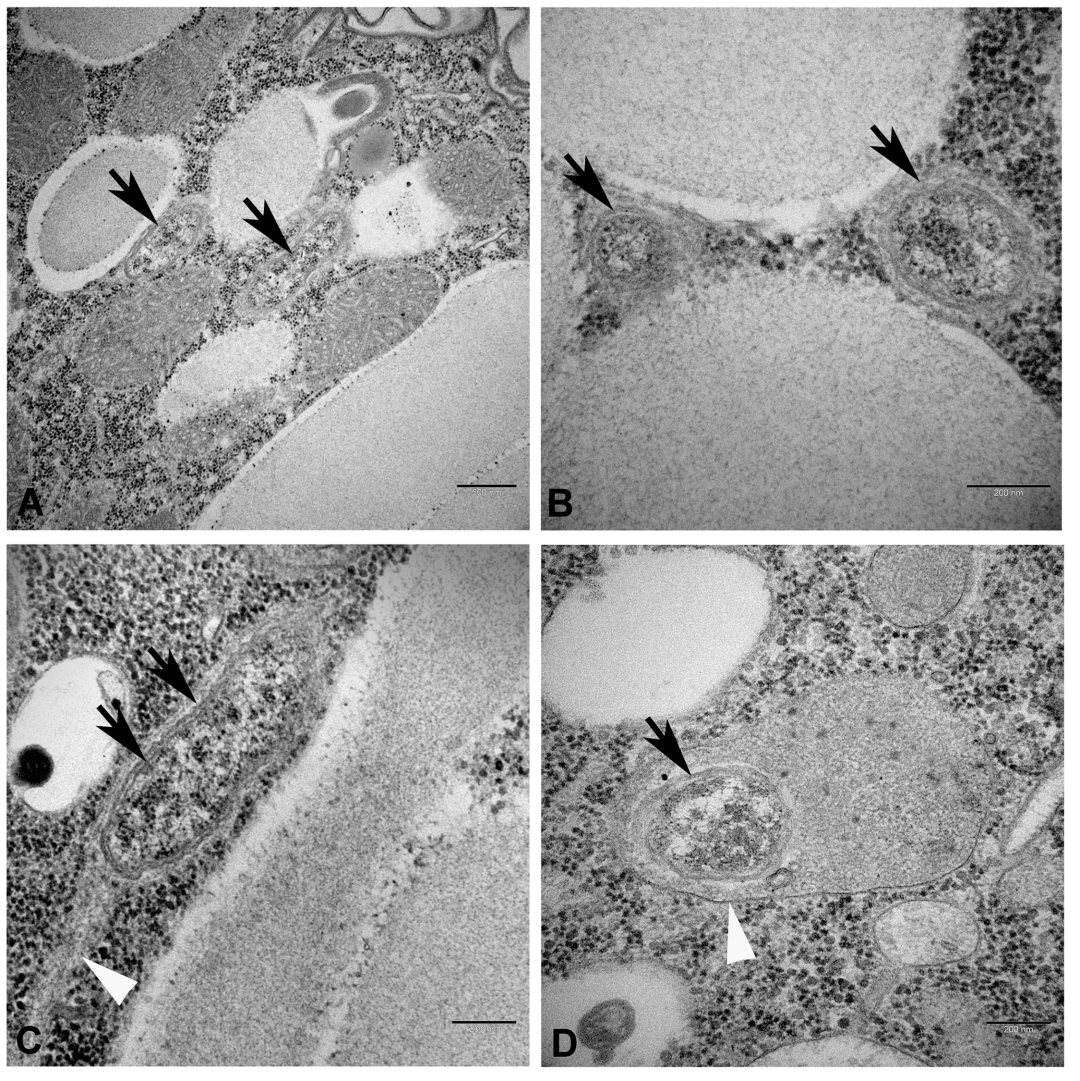
Transmission electron microscopy images of ‘‘*Candidatus* Fokinia solitaria” in longitudinal (A, C) and transverse (B, D) sections. Black arrows point at the bacterial membranes; white arrowheads indicate fibrillar material associated to the endosymbiont (C) and host autolysosome containing the endosymbiont (D). Scale bars: 0.5 μm (A) and 0.2 μm (B, C, D).

**Fig 4:**
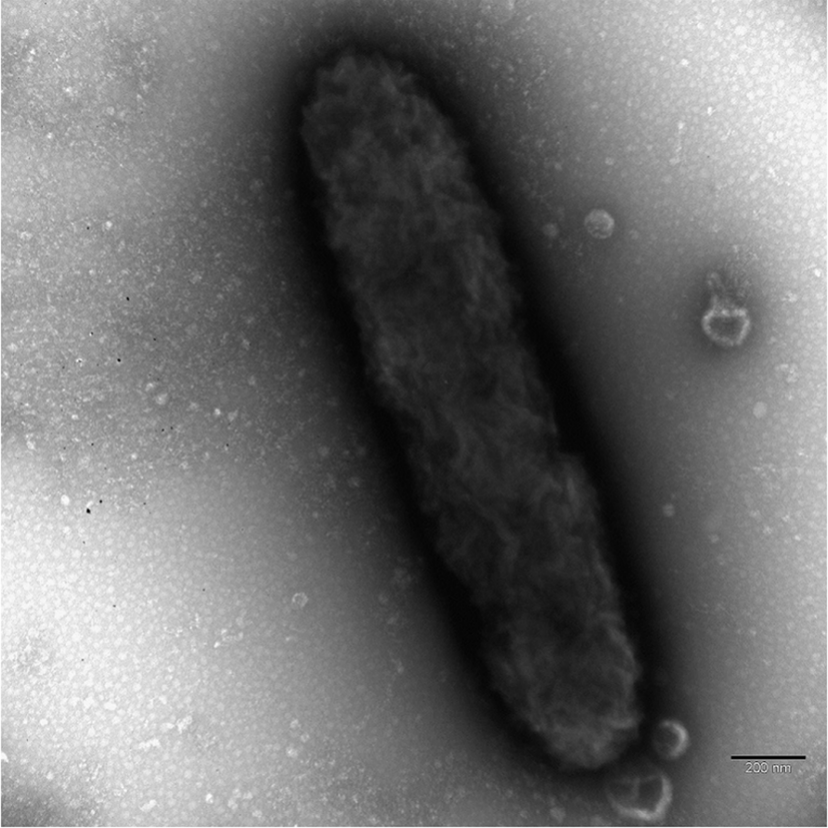
Negative staining of “*Candidatus* Fokinia solitaria”. No flagella are visible. Scale bar: 0.2 μm.

The endosymbionts were located in the host cortex, stratified in a narrow layer in between the trichocysts or just below them (Fig 3A and 3B). Most often, they were oriented parallel to the trichocysts axis and perpendicular to the plasma membrane. In ultrathin sections, endosymbionts appeared as tiny rods, 1.2 μm long and 0.25–0.35 μm wide. They showed a distinct double membrane characteristic of Gram-negative bacteria (Fig 3C). The bacteria never formed clusters and lay naked in the host cytoplasm. Occasionally, dividing forms could be found. No flagella were detected. However, in cross sections bacteria were surrounded by a narrow rim lacking host ribosomes and containing fine fibrils, while in some longitudinal sections, there seemed to be a “tail” of the same material trailing after the endosymbiont (Fig 3C, white arrowhead). However, negative staining demonstrated the absence of flagella (Fig 4). Bacterial ribosomes and nucleoid were quite conspicuous in the bacterial cytoplasm, but other inclusions were rarely observed. The cytoplasm of the infected ciliates was abundant in autolysosomes, most often containing mitochondria; the endosymbionts could be also quite frequently enclosed in autolysosomes (Fig 3D, white arrowhead), sometimes together with mitochondria (not shown).

## Discussion

After the description of “*Ca*. M. mitochondrii” had been published in 2006, the number of species and sequences closely related to “*Ca*. M. mitochondrii” and other members of the family increased remarkably. During the last years, several new genera were discovered and our knowledge of the family *Midichloriaceae* took shape step by step. With the present species description of “*Ca*. Fokinia solitaria” we add a new piece to the puzzle of *Midichloriaceae*.

The families *Midichloriaceae*, *Anaplasmataceae* and *Rickettsiaceae* represent monophyletic clades with high support values (Fig 2). The obtained tree topologies based on bacterial SSU rRNA gene sequences associating *Midichloriaceae* to *Anaplasmataceae* is coherent with all previously published SSU rRNA phylogenies [21,22,25,32,59,60,83,84], except one [51]. Two genome based studies [25,85] showed a closer relationship between *Midichloriaceae* and *Rickettsiaceae*. On the contrary, other recent publications using different sets of species and genes placed *Midichloriaceae* as sister to *Anaplasmataceae*, although with limited support, in agreement with most SSU rRNA trees [32,86]. Further genomic data will be necessary to unambiguously establish evolutionary association among the three families.

The phylogenetic analyses of our data indicated a close association of “*Ca*. Fokinia solitaria” to two different sequences forming a highly supported (99.8% ML; 1.00 BI) monophyletic branch. One of the sequences derives from an uncultured bacterium of a freshwater lake in New York (unpublished; accession number FJ437943), the other one belongs to “*Ca*. Defluviella procrastinata”, an endosymbiotic bacterium inhabiting *P*. *nephridiatum* [60]. Their phylogenetic proximity suggests that these three species might have derived from a common ancestor. Additionally, the occurrence of similar insertions in the SSU rRNA genes of “*Ca*. Fokinia solitaria” and “*Ca*. Defluviella procrastinata”, supports this presumption and suggests that this feature could be a shared derived character of the two genera. The sequence FJ437943 does not share any of the insertions and therefore seems to retain the ancient condition. Nevertheless, as the identity values among the three sequences (84.9–88.5%) are far below the taxonomic threshold for discriminating bacterial genera (sequence similarity of 94.5% or lower, according to [87]), the three sequences belong to different genera. Taking into account their highly supported phylogenetic association, the low sequence identities and the long terminal branches in the phylogenetic analysis, these species appear to be fast evolving.

Up to now, some representatives of *Midichloriaceae*, such as genera “*Ca*. Midichloria”, “*Ca*. Lariskella” and “*Ca*. Bandiella”, have been observed in a great variety of host species and with a worldwide distribution [21,23,64–66,88–91]. Such host species occur both in aquatic and terrestrial habitats. Most likely, the ancestral host species was an aquatic organism indicating at least one event of adaptation to terrestrial animals [61]. Due to the unresolved phylogenetic relationships between the genera of *Midichloriaceae* it is not clear, when and how many times the adaptation to terrestrial animals took place (compare Fig 2 of this work with Fig 4 of [61]). Nevertheless, infection experiments on “*Candidatus* Jidaibacter acanthamoeba” and “*Candidatus* Bandiella woodruffii” proved the possibility of horizontal transfer among aquatic organisms [32,89]. In contrast to these genera, others seem to be represented by few isolates appearing as “stand-alone” branches in the phylogeny of *Midichloriaceae*, not only as “*Ca*. Fokinia”, but also the recently redescribed genus *Lyticum* [59,92–94] as well as “*Ca*. Defluviella” [60] and “*Ca*. Anadelfobacter” [22]. Presently noted “stand-alone” genera of *Midichloriaceae* could be either an indication of poorly sampled organisms, thus underrepresented in GenBank, or fast evolving, highly specialized, real “stand-alone” evolutionary lineages.

Overall, the family *Midichloriaceae* seems to consist of different clades with members showing different evolutionary strategies: widespread and adaptable endosymbiotic bacteria (“*Ca*. Midichloria”, “*Ca*. Lariskella”, and “*Ca*. Bandiella”) on one hand, and fast evolving “stand-alone” symbionts, such as *Lyticum*, “*Ca*. Defluviella”, “*Ca*. Anadelfobacter” and “*Ca*. Fokinia”, on the other hand. To refer to the characteristic of “*Ca*. Fokinia”, represented by an isolated branch, and in accordance with the guidelines of the International Committee of Systematic Bacteriology [67], we propose the name “*Ca*. Fokinia solitaria” in honor to our appreciated colleague Professor Sergei I. Fokin, a prominent specialist in the study of bacterial symbionts of ciliates.

All so far discovered *Midichloriaceae*-endosymbionts of ciliates are rod-shaped but differ significantly in their size, “*Ca*. Fokinia solitaria” being one of the smallest. There are also remarkable differences in the intracellular localization of the endosymbionts. “*Ca*. Fokinia solitaria” and “*Ca*. Cyrtobacter comes” [22] are not surrounded by a host membrane and lie naked in the host cytoplasm, whereas both *Lyticum* species and “*Ca*. Anadelfobacter veles” reside in host vesicles [22,59]. Only “*Ca*. Fokinia solitaria” shows a defined distribution, stratified in a narrow layer in the host cortex. This area is known to be devoid of acid phosphatase (AcPase) activity, indicating the absence of lysosomes and autophagosomes [95,96]. The special localization of endosymbionts between the host trichocysts could be favorable for “*Ca*. Fokinia solitaria”, permitting it to avoid the host defense mechanisms, especially because it is not surrounded by a protective symbiont-containing vacuole. The occurrence of “*Ca*. Fokinia solitaria” in autolysosomes in the inner parts of the cytoplasm seems to support this view. Autophagy is not only a process of degrading macromolecules or organelles to provide nutrition during starvation periods; it is also involved in other biological processes like development and differentiation, cell death as well as immune system and protection against pathogens [97–99]. In mammalian cells, autophagy defends the host cells against pathogenic microbes (xenophagy [100]) like viruses [101], bacteria [102–104] and pathogenic protists [105]. Hence, the loss of “*Ca*. Fokinia solitaria” in one of the sampled *Paramecium* strains may be the result of xenophagy and implies that the endosymbiont is not necessary for the host species and is treated as a pathogen. On the other hand, several pathogens were found to be able to avoid, subvert or even utilize the hosts autophagic machinery for replication [106,107] and egress from the host cell [108].

TEM observation of “*Ca*. Fokinia solitaria” gave no evidence for the existence of flagella (Fig 4) but the occurrence of a narrow rim lacking host ribosomes and containing fine fibrillar material was detected (Fig 3C) and a tail-like structure possibly made out of fibrils has been found in some longitudinal sections. These observations and the distinct distribution of “*Ca*. Fokinia solitaria” inside the host cell indicate the possibility that the bacteria are able to move inside the host cytoplasm, probably by using host actin for the movement [109–112].

The microbial community of wastewater and activated sludge is highly diverse. Due to the enriched abundance of organic matter, wastewater is a perfect milieu for growth of non-pathogenic and pathogenic bacteria and the close association between many different bacteria species increases the development and distribution of virulence and resistance factors (for review see [113]). After passing several steps of clarifying and removing contaminants, the remaining sewage is released into the environment still containing several pathogens [113–115]. Therefore, ciliates play a necessary role in the purification of sewage by supporting the flocculation process [116] and more importantly, as bacterivorous organisms they regulate bacterial biomass and the occurrence of pathogenic bacteria [117,118]. During the process of feeding, they run the risk of being colonized by bacteria [95,119]. Thus, the probability of being infected by potential human or animal pathogens is high in a habitat bearing many different bacteria. Hence, ciliates could play a role as reservoir for pathogens [44] especially in environments like wastewater. Indeed, in some cases, protists have been found to harbor pathogenic bacteria [120–122]. Other potentially pathogenic bacteria have been found in amoeba and ciliates as well [123,124]. “*Ca*. Fokinia solitaria” was found in a *Paramecium* species isolated from a wastewater treatment plant in Rio de Janeiro, Brazil. Up to now, only two other records of bacteria inhabiting ciliates deriving from wastewater are available [60,125], suggesting that the role of ciliates as reservoir for potentially pathogenic bacteria in wastewater may have been overlooked.

A big diversity of *Rickettsiales* not associated with pathogenicity for vertebrates emerged recently [7]. In almost five years of intensive environmental screening for endosymbiotic bacteria in ciliates, eight new species of *Midichloriaceae* corresponding to six new genera have been described, or respectively molecularly characterized for the first time, in ciliate model organisms *Paramecium* and *Euplotes*, i.e. “*Ca*. Defluviella procrastinata”, “*Ca*. Cyrtobacter comes” and “*Ca*. C. zanobii”, “*Ca*. Anadelfobacter veles” [22,60], *Lyticum sinuosum* and *L*. *flagellatum* [59], “*Ca*. Bandiella wodruffii” [89], and this new one, “*Ca*. Fokinia solitaria”. This high rate of new species descriptions indicates a general high abundance of different *Midichloriaceae* species in ciliates and, possibly, in protists. It seems very likely that more descriptions of new *Midichloriaceae* will follow providing us a better understanding of their phylogenetic relationships and host-endosymbiont interactions.

### Description of “*Candidatus* Fokinia solitaria”

“*Candidatus* Fokinia solitaria” (Fo.kiˈni.a so.li. taˈri.a; N.L. fem. n. *Fokinia*, in honor of Professor Sergei I. Fokin; N.L. adj. *solitarius*, solitary, lonely). Short rod-like bacterium (1.2 x 0.25–0.35 μm in size). Cytoplasmic endosymbiont of the ciliate *Paramecium* sp. strain Rio ETE_ALG 3VII (Oligohymenophorea, Ciliophora). Basis of assignment: SSU rRNA gene sequence (accession number: KM497527) and positive match with the specific FISH oligonucleotide probes Fokinia_198 (5'-CTTGTAGTGACATTGCTGC-3'), Fokinia_434 (5'-ATTATCATCCCTACCAAAAGAG-3') and Fokinia_1250 (5'-ACCCTGTTGCAGCCTTCT-3'). Belongs to *Midichloriaceae* family in the order *Rickettsiales* (*Alphaproteobacteria*). Identified in *Paramecium* sp. strain Rio ETE_ALG 3VII isolated from a wastewater treatment plant in Rio de Janeiro (Brazil). Uncultured thus far.

## Supporting Information

S1 Alignment Alignment of the bacterial SSU rRNA gene sequence of “*Ca*. Fokinia solitaria”. 22 closely related sequences of the *Midichloriaceae* family, 17 members of the *Anaplasmataceae* and *Rickettsiaceae* and 10 other *Alphaproteobacteria* representing the outgroup were aligned with “*Ca*. Fokinia solitaria” to perform phylogenetic analyses. The alignment was trimmed at both ends to reach the length of the shortest sequence on each side, the resulting 1,568 nucleotide columns are presented here.
(S1_ALIGNMENT)

S1 Table Obtained model parameter values of the executed GTR + I + G model according to the performed MrBayes analysis of our sequence alignment. The parameters are given as total tree length (TL), reversible substitution rates (r(A<->C), r(A<->G), etc), stationary state frequencies of the four bases (pi(A), pi(C), etc), the shape of the gamma distribution of rate variation across sites (alpha), and the proportion of invariable sites (pinvar). The estimated sampling size (ESS) is shown as minimal (minESS) and average (avgESS) values. PSRF stands for potential scale reduction factor.
(DOCX)
