# Supplementary material for: “Candidatus Fokinia solitaria”, a Novel “Stand-Alone” Symbiotic Lineage of Midichloriaceae (Rickettsiales)
Source: PLoS One. 2016 Jan 5;11(1):e0145743. doi: 10.1371/journal.pone.0145743 (PMC4701390; doi:10.1371/journal.pone.0145743)
Supplement: S1 Table — The parameters are given as total tree length (TL), reversible substitution rates (r(A<->C), r(A<->G), etc), stationary state frequencies of the four bases (pi(A), pi(C), etc), the shape of the gamma distribution of rate variation across sites (alpha), and the proportion of invariable sites (pinvar). The estimated sampling size (ESS) is shown as minimal (minESS) and average (avgESS) values. PSRF stands for potential scale reduction factor. (DOCX) [file pone.0145743.s002.docx]

“*Candidatus* Fokinia solitaria”, a novel “stand-alone” symbiotic lineage of *Midichloriaceae* (*Rickettsiales*)

Journal: PloS ONE

Authors: Franziska Szokoli, Elena Sabaneyeva, Michele Castelli, Sascha Krenek, Martina Schrallhammer, Carlos A. G. Soares, Inacio D. da Silva-Neto, Thomas U. Berendonk, Giulio Petroni*

*Corresponding author. Mailing address: Biology Department, Protistology-Zoology Unit, University of Pisa, Via A.Volta 4, 56126 Pisa, Italy. Phone: 39 050 2211384. Fax: 39 050 2211393. E-mail: gpetroni@biologia.unipi.it.

Sequences 50

Columns 1,568

Model selected GTR + I + G

(by jModelTest)

| Parameter | Mean | Variance | Lower | Upper | Median | minESS | avgESS | PSRF |
| --- | --- | --- | --- | --- | --- | --- | --- | --- |
| TL | 5.051 | 0.050 | 4.613 | 5.479 | 5.043 | 1,356.721 | 1,452.907 | 1.001 |
| r(A<->C) | 0.052 | 0.000 | 0.041 | 0.064 | 0.051 | 750.477 | 816.337 | 1.000 |
| r(A<->G) | 0.318 | 0.000 | 0.287 | 0.349 | 0.317 | 418.178 | 436.562 | 1.001 |
| r(A<->T) | 0.112 | 0.000 | 0.097 | 0.126 | 0.112 | 736.866 | 754.175 | 1.000 |
| r(C<->G) | 0.058 | 0.000 | 0.047 | 0.069 | 0.057 | 821.370 | 881.743 | 1.001 |
| r(C<->T) | 0.407 | 0.000 | 0.374 | 0.443 | 0.407 | 420.833 | 435.508 | 1.000 |
| r(G<->T) | 0.053 | 0.000 | 0.043 | 0.063 | 0.053 | 694.669 | 814.514 | 1.000 |
| pi(A) | 0.275 | 0.000 | 0.259 | 0.291 | 0.275 | 615.099 | 726.014 | 1.002 |
| pi(C) | 0.194 | 0.000 | 0.178 | 0.208 | 0.193 | 436.082 | 577.713 | 1.000 |
| pi(G) | 0.261 | 0.000 | 0.244 | 0.278 | 0.261 | 589.913 | 654.630 | 1.000 |
| pi(T) | 0.271 | 0.000 | 0.255 | 0.288 | 0.270 | 577.508 | 671.882 | 1.000 |
| alpha | 0.676 | 0.003 | 0.561 | 0.791 | 0.674 | 986.665 | 1,040.115 | 1.000 |
| pinvar | 0.371 | 0.001 | 0.327 | 0.412 | 0.372 | 1048.449 | 1,127.873 | 1.000 |

**S1 Table. Obtained model parameter values of the executed GTR + I + G model according to the performed MrBayes analysis of our sequence alignment.** The parameters are given as total tree length (TL), reversible substitution rates (r(A<->C), r(A<->G), etc), stationary state frequencies of the four bases (pi(A), pi(C), etc), the shape of the gamma distribution of rate variation across sites (alpha), and the proportion of invariable sites (pinvar). The estimated sampling size (ESS) is shown as minimal (minESS) and average (avgESS) values. PSRF stands for potential scale reduction factor.
